# Supplementary material for: Structural plasticity of mumps virus nucleocapsids with cryo-EM structures
Source: Commun Biol. 2021 Jul 2;4:833. doi: 10.1038/s42003-021-02362-0 (PMC8253768; doi:10.1038/s42003-021-02362-0)
Supplement: Supplementary file 2 — Supplementary Information [file 42003_2021_2362_MOESM2_ESM.pdf]

Supplementary Materials for

Structural plasticity of mumps virus nucleocapsids with cryo-EM structures

Authors: Hong Shan[1,2*], Xin Su[1*], Tianhao Li[1*], Yuqi Qin[1*], Na Zhang[1,3], Liuyan Yang[4,5], Linsha Ma[6], Yun Bai[6], Lei Qi[2], Yunhui Liu[1], Qing-Tao Shen[1,2✉]

Affiliations:

[1]iHuman Institute and School of Life Science and Technology, ShanghaiTech University, Shanghai 201210, China.

[2]Laboratory for Marine Biology and Biotechnology, Qingdao National Laboratory for Marine Science and Technology, Qingdao 266237, China.

[3]University of Chinese Academy of Sciences, Beijing 100049, China.

[4]State Key Laboratory of Microbial Technology, Marine Biotechnology Research Center, Shandong University, Qingdao 266237, China

[5]College of Marine Life Sciences, Ocean University of China, Qingdao 266003, China.

[6]School of Life Science and Technology, ShanghaiTech University, Shanghai 201210, China.

[*]These authors contributed equally: Hong Shan, Xin Su, Tianhao Li, Yuqi Qin

✉ Correspondence to: Qing-Tao Shen (shenqt@shanghaitech.edu.cn)

The PDF file includes:

Supplementary Figs. 1 to 8

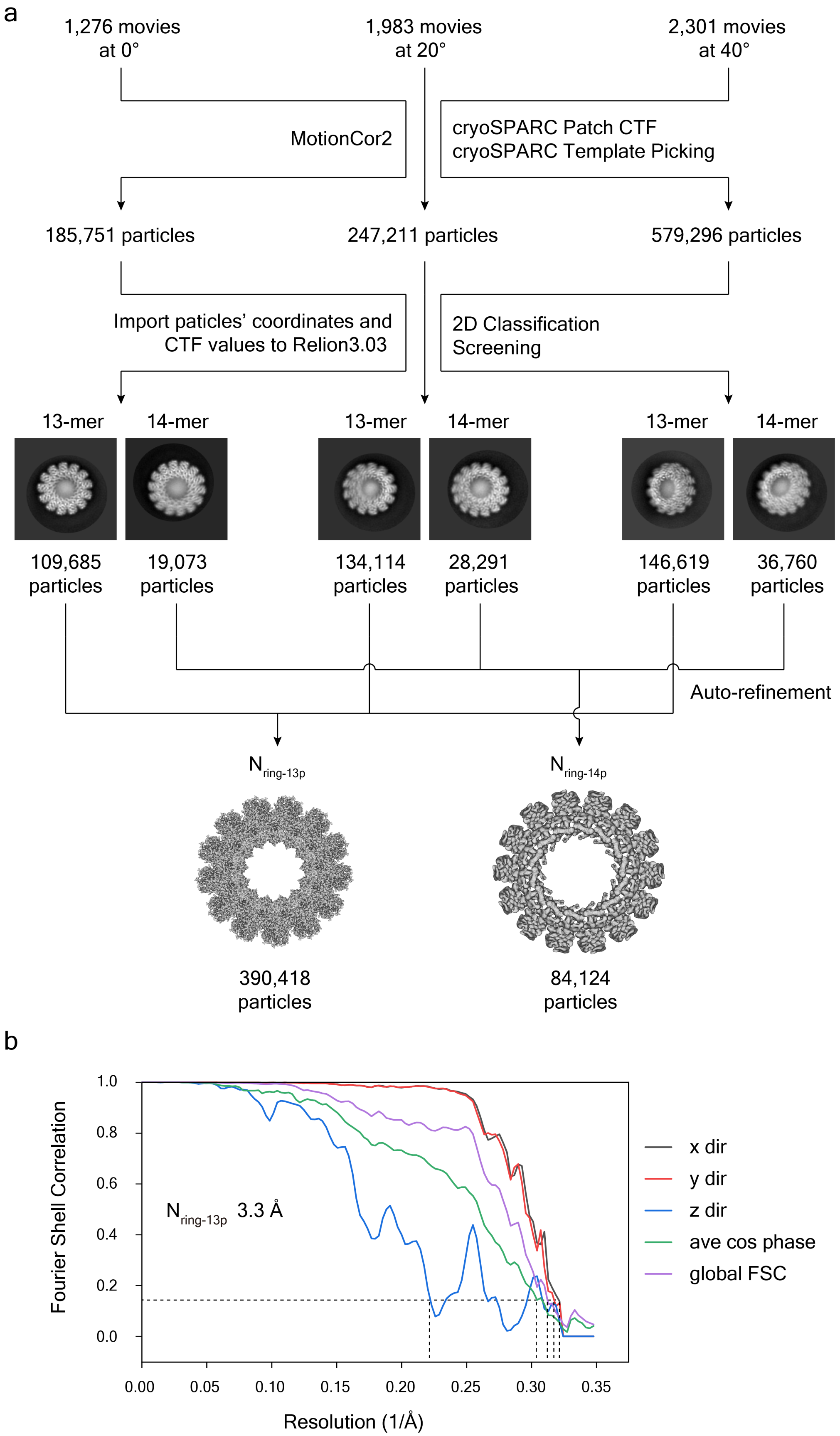
a
1,276 movies at 0°
1,983 movies at 20°
2,301 movies at 40°
MotionCor2
cryoSPARC Patch CTF
cryoSPARC Template Picking
185,751 particles
247,211 particles
579,296 particles
Import paticles' coordinates and CTF values to Relion3.03
2D Classification Screening
13-mer
14-mer
13-mer
14-mer
13-mer
14-mer
109,685 particles
19,073 particles
134,114 particles
28,291 particles
146,619 particles
36,760 particles
Auto-refinement
N ring-13p
N ring-14p
390,418 particles
84,124 particles
b
Fourier Shell Correlation
1.0
0.8
0.6
0.4
0.2
0.0
N ring-13p 3.3 Å
0.00
0.05
0.10
0.15
0.20
0.25
0.30
0.35
Resolution (1/Å)
x dir
y dir
z dir
ave cos phase
global FSC

Supplementary Fig. 1 The flow chart for 3D reconstruction of MuV N-RNA rings, and the FSC curve. a The flow chart for 3D reconstruction of MuV N-RNA rings. **b** 3D FSC curve of MuV $N_{ring\text{-}13p}$.

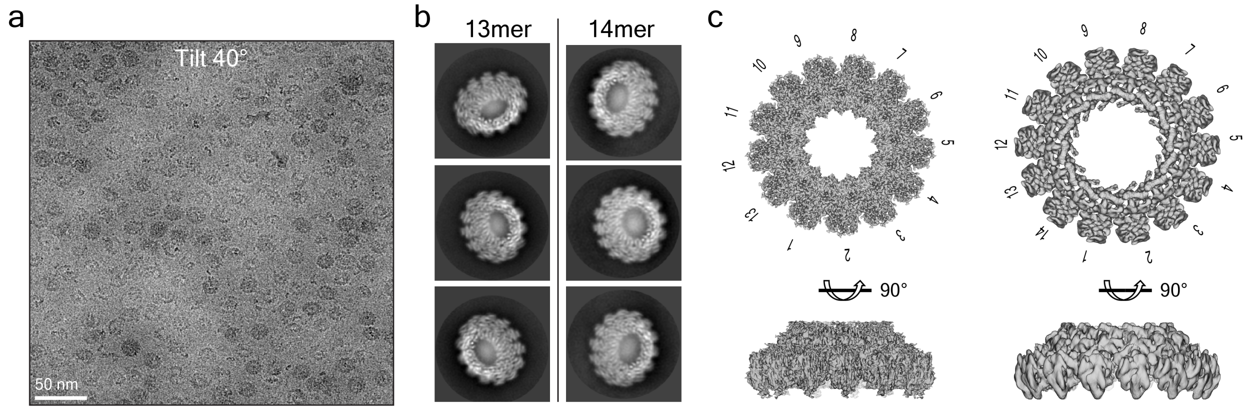

Supplementary Fig. 2 3D reconstruction of MuV N-RNA rings. **a** A typical EM image of MuV N-RNA rings collected with the tilting angle at 40°. **b** 2D classification average of MuV N-RNA rings with the tilting angle at 40°. **c** 3D reconstructs of MuV $N_{ring-13p}$ and $N_{ring-14p}$. Both top and side views are shown for $N_{ring-13p}$ (left) and $N_{ring-14p}$ (right).

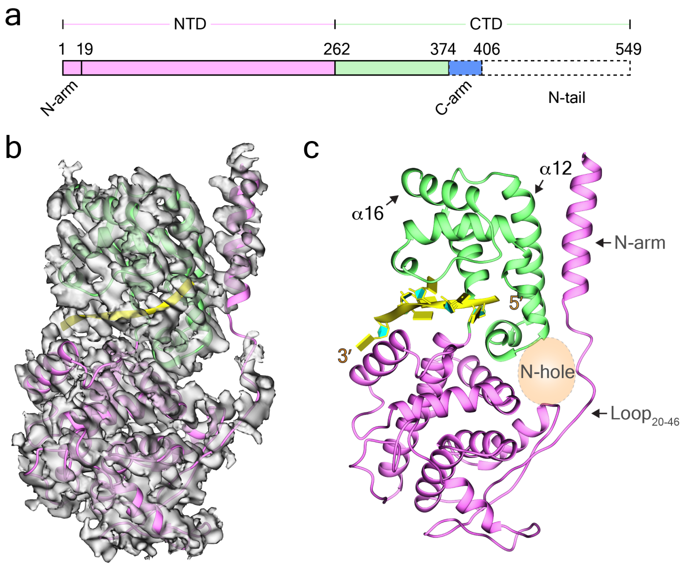

Supplementary Fig. 3 Atomic model of one protomer in $N_{ring-13p}$. a The domain organization of MuV nucleoprotein. NTD and CTD are colored in pink and green respectively, and the C-arm is emphasized in blue. **b** EM density of one protomer of MuV $N_{ring-13p}$ and the docked atomic model. **c** The atomic model of one $N_{ring-13p}$ protomer. C-arm and N-tail are invisible in the model.

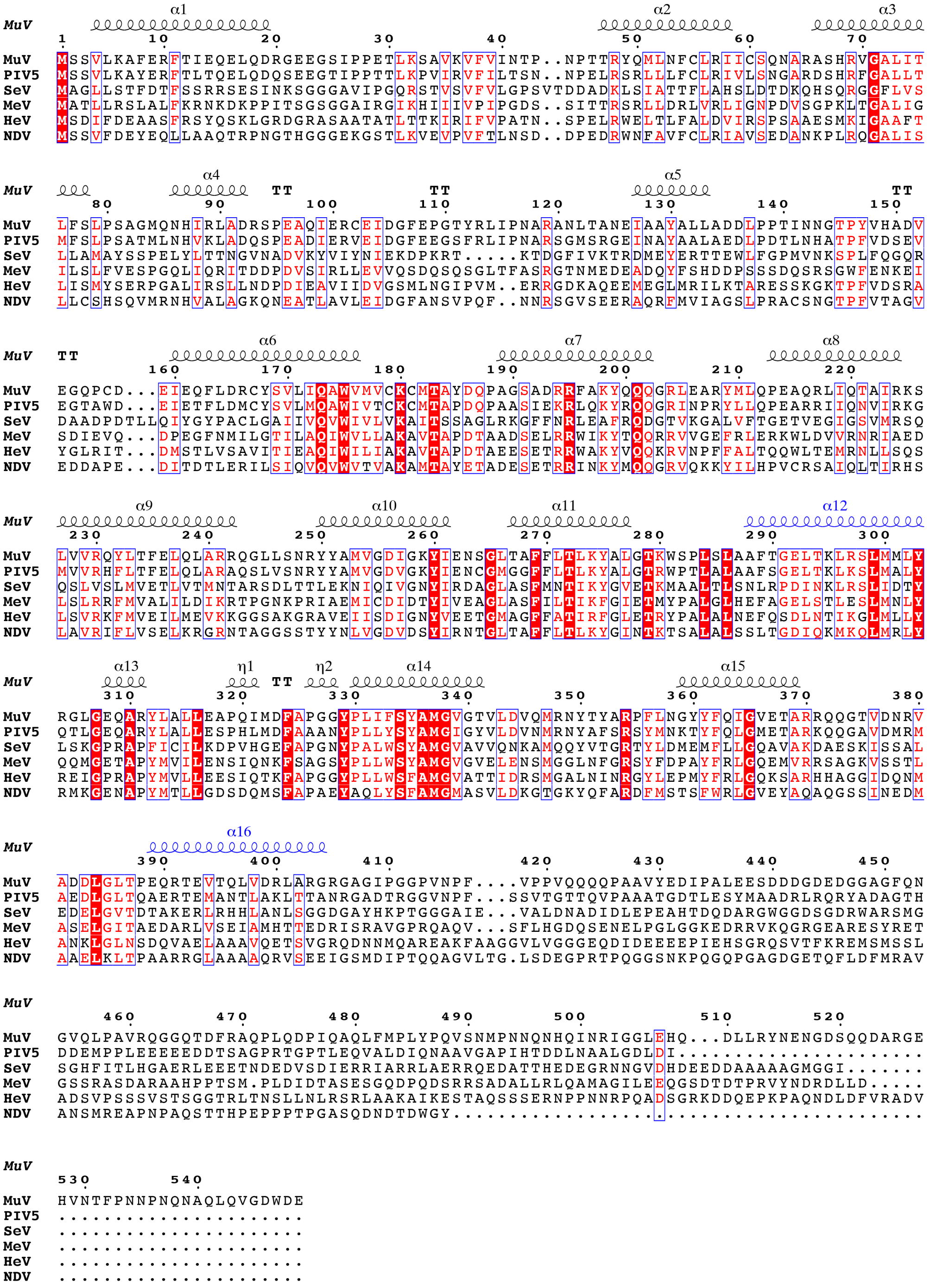

Supplementary Fig. 4 Sequences alignment of nucleoproteins from different species in the order of *Mononegavirales*. The α1–α16 helices from the atomic model of MuV $N_{ring\text{-}13p}$ are labeled on the respective sequences. The α12 and α16 helices are highlighted in blue.

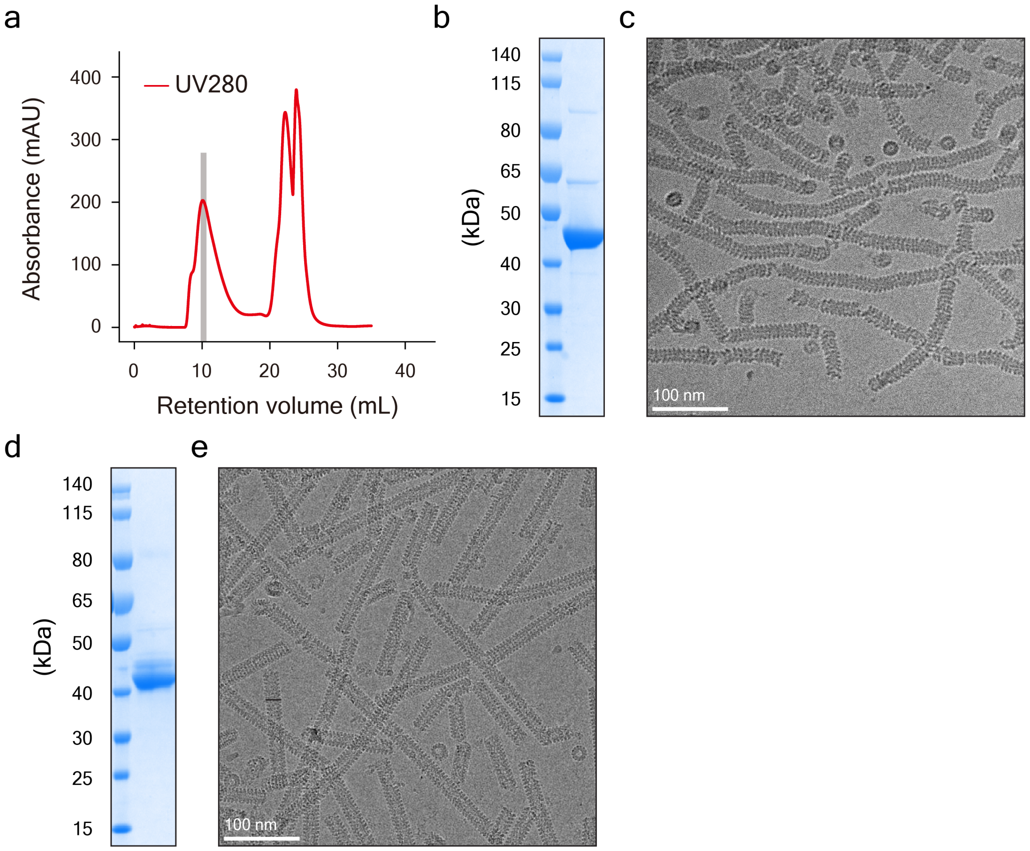

Supplementary Fig. 5 Filaments formed by MuV $N_{\Delta N\text{-tail}}$ and trypsin digested N_{WT}. a Gel filtration chromatography of MuV $N_{\Delta N\text{-tail}}$. The fraction at ~10.0 mL was selected for SDS-PAGE analysis and cryo-EM analysis. **b**, **c** SDS-PAGE gel and cryo-EM image of MuV $N_{\Delta N\text{-tail}}$. **d, e** SDS-PAGE gel and cryo-EM image of MuV N_{WT} after trypsin digestion.

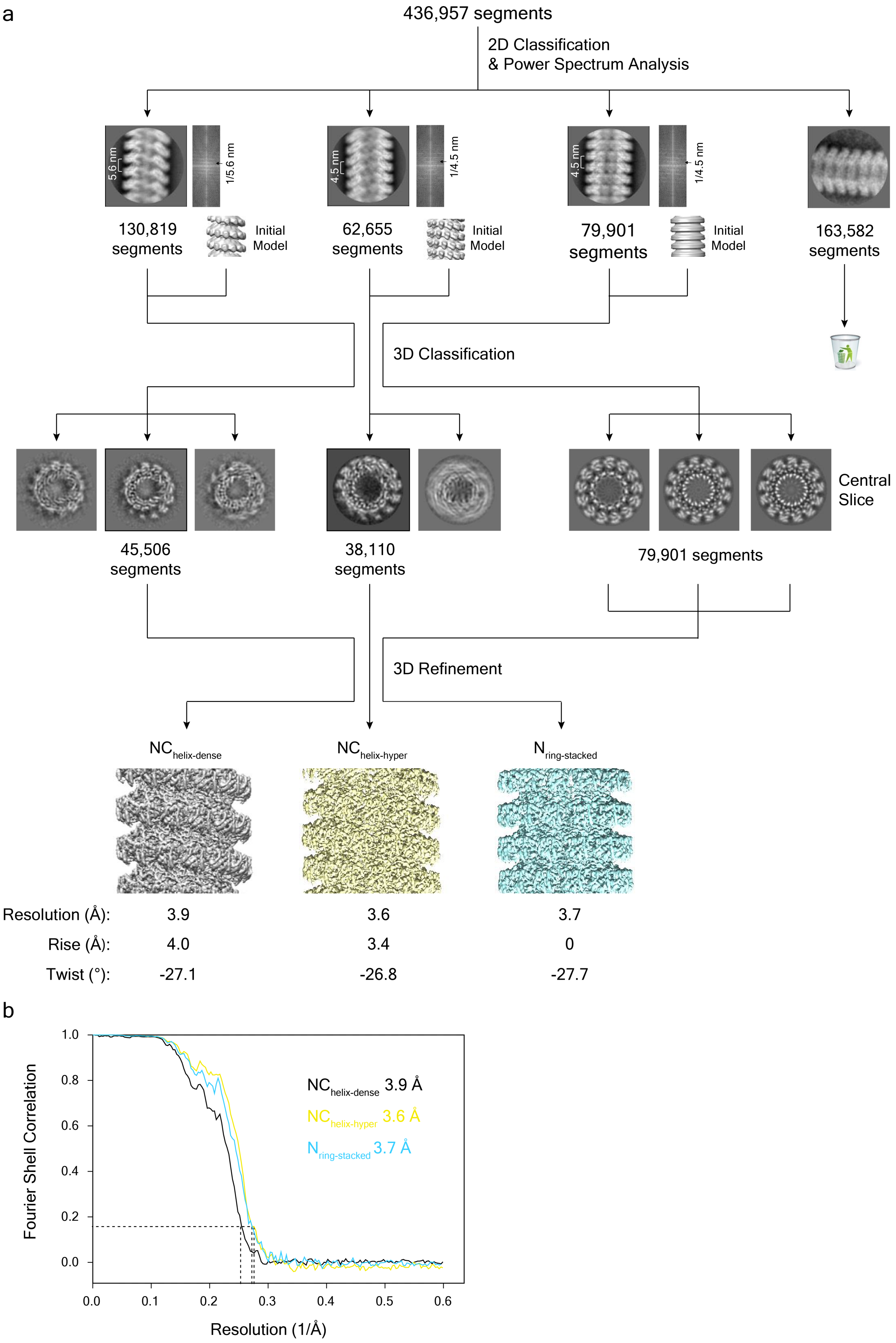
a
436,957 segments
2D Classification
& Power Spectrum Analysis
5.6 nm
1/5.6 nm
4.5 nm
1/4.5 nm
4.5 nm
1/4.5 nm
130,819 segments
Initial Model
62,655 segments
Initial Model
79,901 segments
Initial Model
163,582 segments
3D Classification
Central Slice
45,506 segments
38,110 segments
79,901 segments
3D Refinement
NChelix-dense
NChelix-hyper
Nring-stacked
Resolution (Å): 3.9 3.6 3.7
Rise (Å): 4.0 3.4 0
Twist (°): -27.1 -26.8 -27.7
b
Fourier Shell Correlation
Resolution (1/Å)
NChelix-dense 3.9 Å
NChelix-hyper 3.6 Å
Nring-stacked 3.7 Å

Supplementary Fig. 6 A flow chart for 3D reconstruction of MuV $NC_{helix\text{-}dense}$, $NC_{helix\text{-}hyper}$ and $N_{ring\text{-}stacked}$ and the respective FSC curves. a The flow chart for 3D reconstruction of MuV $NC_{helix\text{-}dense}$, $NC_{helix\text{-}hyper}$, and $N_{ring\text{-}stacked}$. **b** Gold-standard FSC curves of MuV $NC_{helix\text{-}dense}$, $NC_{helix\text{-}hyper}$, and $N_{ring\text{-}stacked}$.

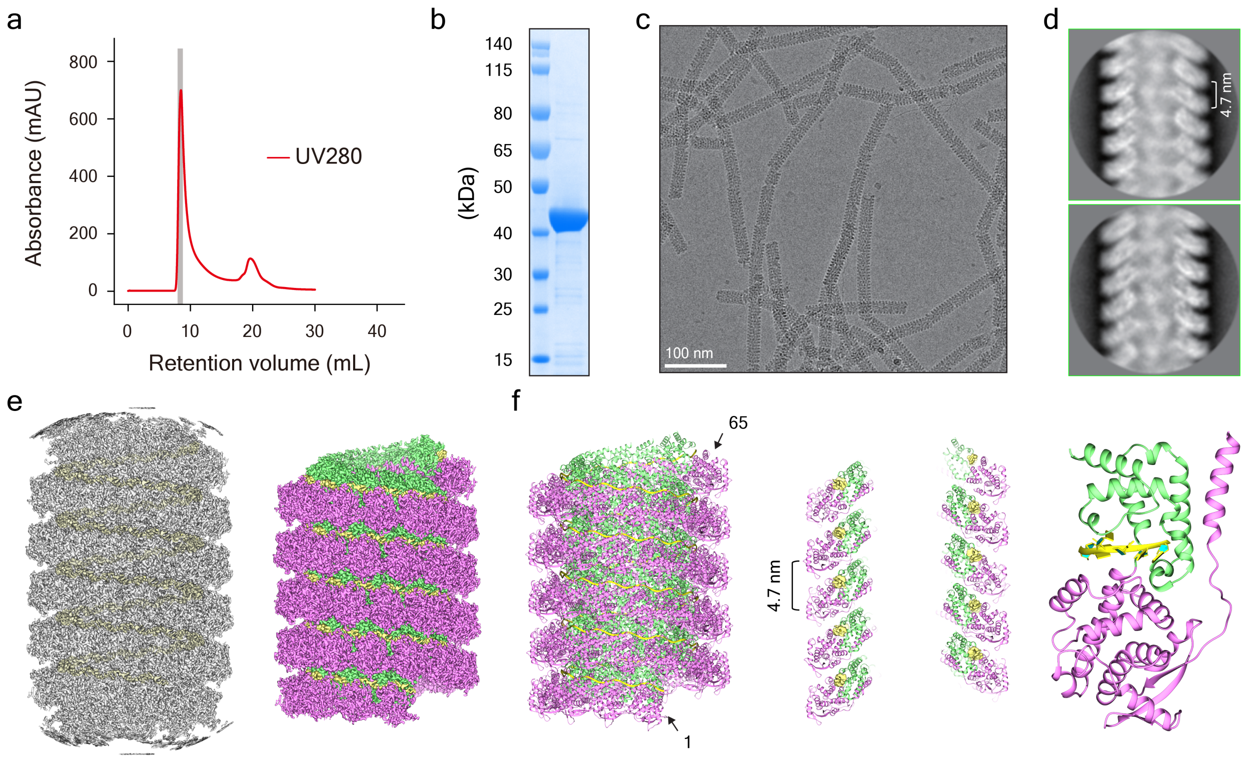

Supplementary Fig. 7 Hyperdense MuV NC$_{helix\text{-}\Delta arm}$. **a** Gel filtration chromatography of MuV N$_{\Delta arm}$. Fraction at ~9.0 mL was selected for SDS-PAGE analysis and cryo-EM analysis. **b** SDS-PAGE gel of MuV N$_{\Delta arm}$. **c** Cryo-EM image of MuV N$_{\Delta arm}$. **d** 2D class average of the segments of MuV N$_{\Delta arm}$. Helical pitch at 4.7 nm is labeled. **e** 3D reconstruction of MuV NC$_{helix\text{-}\Delta arm}$. **f** The atomic model of MuV NC$_{helix\text{-}\Delta arm}$.

a

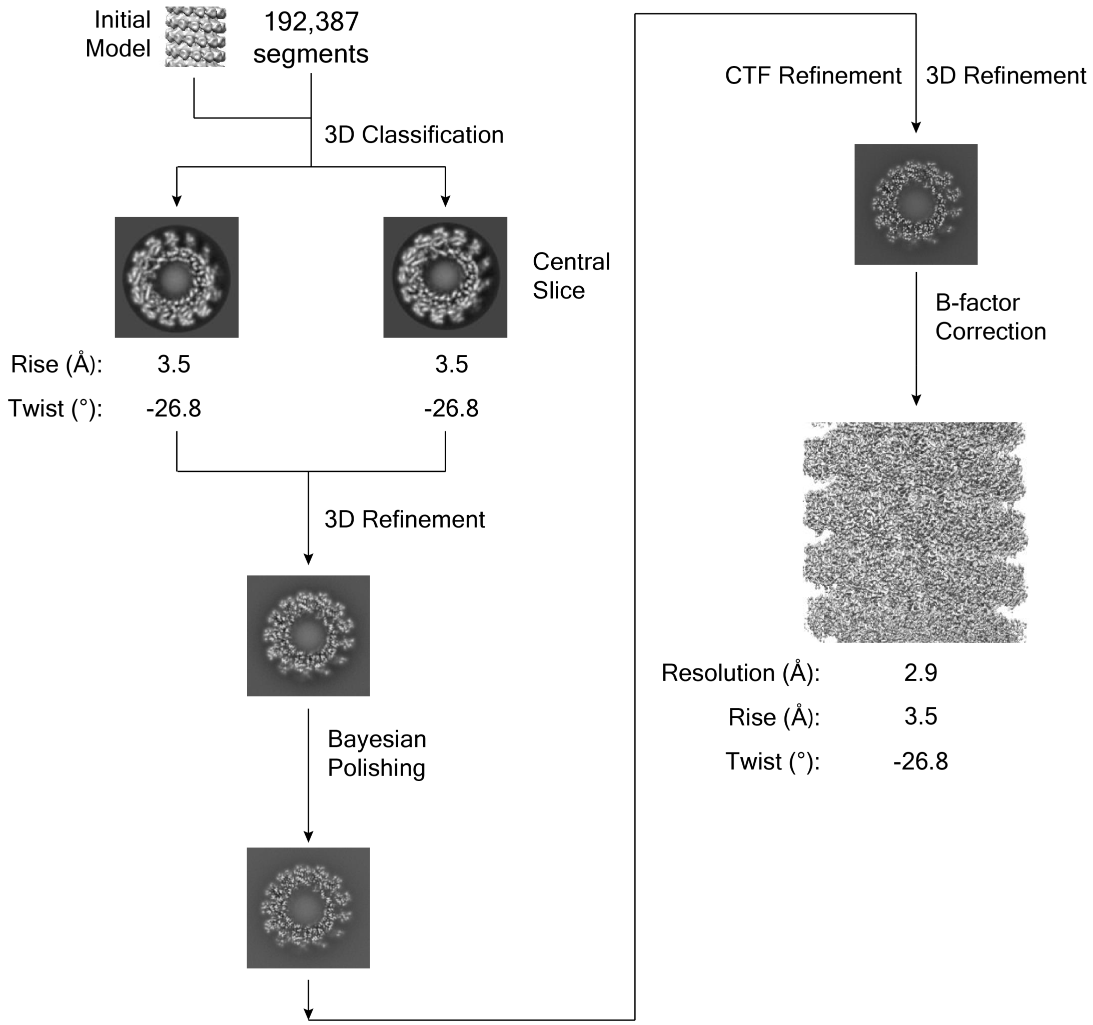

Initial Model
192,387 segments
3D Classification
Central Slice
Rise (Å): 3.5 3.5
Twist (°): -26.8 -26.8
3D Refinement
Bayesian Polishing
CTF Refinement
3D Refinement
B-factor Correction
Resolution (Å): 2.9
Rise (Å): 3.5
Twist (°): -26.8

b

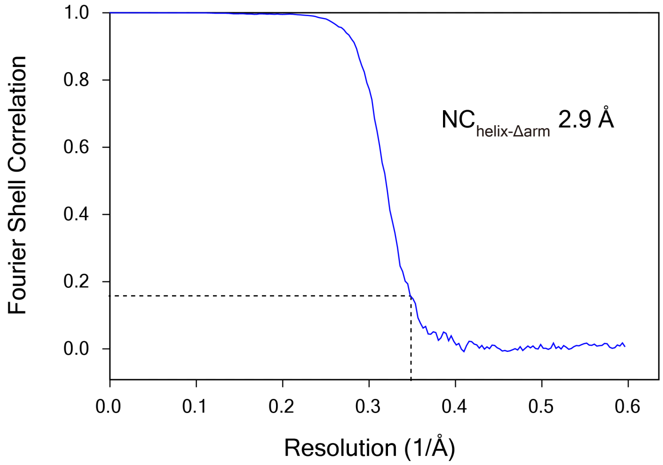

Fourier Shell Correlation
1.0
0.8
0.6
0.4
0.2
0.0
0.0 0.1 0.2 0.3 0.4 0.5 0.6
Resolution (1/Å)
NChelix-Δarm 2.9 Å

Supplementary Fig. 8 The flow chart for 3D reconstruction of $NC_{helix\text{-}\Delta arm}$ and the FSC curve. **a** The flow chart for 3D reconstruction of MuV $NC_{helix\text{-}\Delta arm}$. **b** Gold-standard FSC curve of MuV $NC_{helix\text{-}\Delta arm}$.
