# Supplementary material for: Structural plasticity of mumps virus nucleocapsids with cryo-EM structures
Source: Commun Biol. 2021 Jul 2;4:833. doi: 10.1038/s42003-021-02362-0 (PMC8253768; doi:10.1038/s42003-021-02362-0)
Supplement: Supplementary file 3 — Descriptions of Additional Supplementary Files [file 42003_2021_2362_MOESM3_ESM.pdf]

Descriptions of Additional Supplementary Files

Supplementary Movie 1
Description: Morphing from MuV Nring-stacked to NChelix-dense.

Supplementary Movie 2
Description: Morphing between MuV NChelix-dense and NChelix-hyper.
